# Single nucleotide polymorphism information estimates breed and variety composition ratio in food

**DOI:** 10.1016/j.crfs.2026.101312

**Published:** 2026-01-13

**Authors:** Cheng-En Tan, Ilias Tagkopoulos

**Affiliations:** a Department of Computer Science, University of California, Davis, Davis, CA, 95616, United States; b Genome Center, University of California, Davis, Davis, CA, 95616, United States; c USDA/NSF AI Institute for Next Generation Food Systems (AIFS), University of California, Davis, Davis, CA, 95616, United States

## Abstract

The quality of food products can be influenced by the breed or variety of origin, as well as the composition ratios in mixtures of breeds or varieties. We present a method to estimate the breed or variety composition ratio in food samples using single-nucleotide polymorphism (SNP) allele frequency data and a non-negative least squares (NNLS) optimization approach. To evaluate the method's performance, we simulated two datasets (cow and cacao) containing simulated samples with specified breed or variety composition ratios, then compared the predicted ratios to the actual values. Results show that the method estimates the composition ratios of breeds and varieties with significantly lower average absolute error than a uniform probability baseline (4.1 % vs 24.6 % for cows, p-value = 1.9 × 10^−17^; and 11.8 % vs 24.6 % for cacao, p-value = 1.1 × 10^−8^). Additionally, the accuracy of identifying the majority breed or variety in a sample is also significantly higher than assuming equal probability of breed mixing (92 % vs 28 % for cows and 72 % vs 28 % for cacao). The corresponding code for the breed or variety composition ratio estimation is available in the Github repository: (https://github.com/IBPA/NNLS-SNP).

## Highlights

• A novel tool is developed to estimate breed or variety composition ratios in food using SNP allele frequency data.
• The tool verified both the composition ratio and the majority breed or variety in simulated mixed food samples and can be applied to real datasets in the future.
• The method requires only the reference SNP allele frequencies of the breed or variety to be evaluated and does not require model training.

## Graphical abstract

**Figure:**
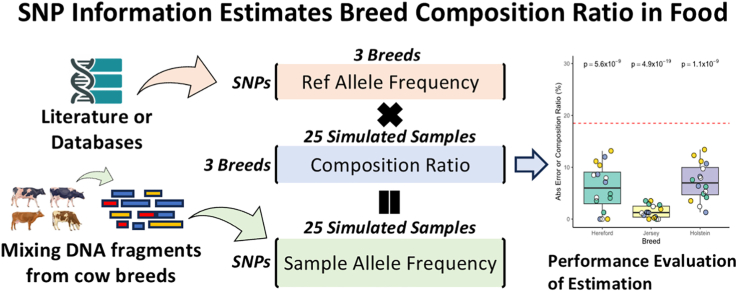

## 1 Introduction

The quality of processed food products – both animal and plant-based – can be affected by their origin breeds or varieties. Different breeds or varieties possess distinct Single Nucleotide Polymorphism (SNP) genotypes, which contribute to phenotypic variation and, consequently, differences in food properties. For example, the chemical compound composition of milk differs between Holstein and Jersey cows (Lim et al., 2020; Czerniewicz et al., 2006; Carroll et al., 2006; White et al., 2001), and these differences directly impact cheese production (Auldist et al., 2004; Bland, 2015; Capper and Cady, 2012); Wines from different grape varieties have different chemical composition profiles (Guth, 1998; Li et al., 2011), and they affect qualities like aroma and flavor (Styger et al., 2011). Likewise, cacao bean varieties such as Criollo and Forastero have different volatile compound profiles (Quelal et al., 2023; Wahyuni et al., 2021), which in turn influence flavor and overall quality of chocolate (Wahyuni et al., 2021).

Mixing ingredients from different animal breeds or plant varieties during food production can combine their chemical, flavor, and odor characteristics, potentially improving quality. For example, wine blending is a widely used approach that combines different varieties of wines (e.g., Cabernet Sauvignon, Merlot, and Cabernet franc) in various proportions to yield products with new flavor profiles (Hopfer et al., 2012; Dooley et al., 2012; Ferrier and Block, 2001). Similarly, adding Jersey cow milk to Holstein milk alters milk properties, such as increasing fat globule size and decreasing casein micelle size (Bland et al., 2015). Blending different varieties of cacao beans is also common in cacao-related studies to modulate flavor and bioactive components (Żyżelewicz et al., 2018; Barrientos et al., 2019; Graziani de et al., 2002). Therefore, estimating the breed or variety composition ratios in food products can be valuable for optimizing quality attributes like odor, flavor, and nutrient content.

Despite this importance, limited tools exist to estimate breed or variety composition ratios in food products. Some methods have been developed to determine species composition in food products (e.g., the proportions of different meat species in sausage) using DNA sequencing data (Ripp et al., 2014; Kobus et al., 2020; Hellmann et al., 2020). Other studies have estimated the breed composition of individual animals from genotype data for ancestry tracing (Alexander and Lange, 2011; Wang et al., 2020; He et al., 2018). However, no tool has been reported that directly estimates the breed or variety composition ratio in food containing arbitrary mixtures of breeds or varieties.

Recently, several general food composition databases have been developed (Youn et al., 2024) to support computational food science research. In addition to these general-purpose resources, more specialized datasets — such as the Bovine Genome Variation Database (BGVD (Chen et al., 2020)) — have also been released, containing breed-specific SNP information. These newly available data enable the estimation of food properties such as flavor (Gunning and Tagkopoulos, 2025; Tan et al., 2024), micronutrient content (Naravane and Tagkopoulos, 2023), and breed or variety composition ratios in complex food mixtures. They also support machine learning and predictive modeling aimed at understanding and improving both the quality and quantity of food production (Youn et al., 2020; Oliveira et al., 2021).

In this study, we propose a tool to fill that gap by estimating the breed or variety composition ratios in food products based on their SNP allele frequencies evaluated from the corresponding DNA sequencing (DNA-seq) data. This tool takes two inputs, including the genotyping information of food product samples in VCF format (Danecek et al., 2011) and reference SNP allele frequency profiles of selected breeds or varieties. It assumes that the SNP allele frequency profile of a sample is a linear combination of the allele frequency profiles of each selected breed or variety. The composition ratios are then solved by setting a linear system and applying an NNLS solver. The performance of this tool is evaluated by comparing the estimated composition ratios and the actual value of two simulated food datasets.

## 2 Datasets and methods

### 2.1 DNA-Seq data Simulation

**Fig. 1:**
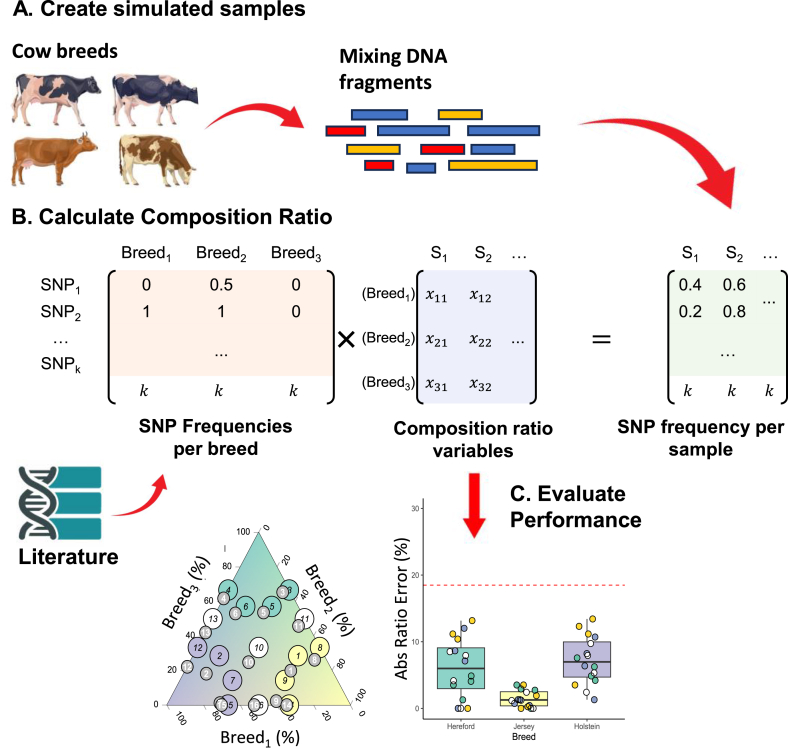
Schematic of the breed and variety composition ratio estimation tool. **A.** Simulation of the food samples mixed with different breeds or variety composition ratios. **B.** SNP reference data curation. The SNP allele frequencies of breeds or varieties are collected from databases or published studies. **C.** Breed or variety composition ratio estimation using an NNLS solver.

To evaluate the performance of the tool, we created two simulated DNA-seq datasets *in silico* that contain simulated samples mixed from different cow breeds and cacao varieties, respectively (Fig. 1A). These two datasets simulated the milk and chocolate production by mixing raw milk and cacao bean powder. We downloaded the whole-genome sequences from NCBI genome database (Kitts et al., 2016) of selected three cow breeds (Holstein, Jersey, and Hereford) and three cacao varieties (IMC 67, Pound 7, SCA 6) (Table 1), and then applied the tool ART (Huang et al., 2012) (version 2016-06-05) to simulate DNA-seq data in FASTQ (Fields et al., 2010) format for each breed or variety at three different coverages (5X, 10X, and 20X). Simulation parameters (HiSeq 2500 sequencing system, paired-end reads, average fragment length of 288 base pairs, and standard deviation of fragment length of 69 base pairs) were chosen to mimic real sequencing data. Finally, for each simulated dataset, 25 simulated samples with various breed or variety composition ratios were generated by concatenating the DNA-seq data with different coverages (Table 2).

**Table 1:** Selected breeds or varieties of two food species for DNA-seq data simulation, and the corresponding reference genome used for alignment in SNP detection. DNA-seq data for each breed or variety were generated at different coverages (5X, 10X, and 20X), and then DNA-seq data with different coverages were combined to simulate the food samples with different breed or variety composition ratios.

Species	Breed or Variety	NCBI Genome Label and ID (Size)	Reference Genome Used (Size)
Cattle	Hereford	GCF_002263795.3 (2.8 Gb)	UMD 3.1.1 (Zimin et al., 2009) (GCA_000003055.5) (2.7 Gb)
	Jersey	GCA_021234555.1 (2.6 Gb)	
	Holstein	GCA_021347905.1 (2.7 Gb)	
Cacao	IMC 67	GCA_036851125.1 (380.9 Mb)	Martina 1–6 (Motamayor et al., 2013) (GCA_000403535.1) (346 Mb)
	Pound 7	GCA_036851155.1 (405.5 Mb)	
	SCA 6	GCA_036851095.1 (382.4 Mb)

**Table 2:** Breed or variety mixing ratios for the 25 simulated samples. DNA-seq data from three selected breeds or varieties with different coverages were combined to simulate different breed or variety composition ratios. The mix ratios apply to both the cow breed and cacao variety datasets.

Samples	Selected Data for Concatenation (Cow/Cacao)			Expected Mix Ratio (Cow/Cacao)		
	Hereford/IMC 67	Jersey/Pound 7	Holstein/SCA6	Hereford/IMC 67	Jersey/Pound 7	Holstein/SCA 6
Sample1	10X	20X	5X	28.57 %	57.14 %	14.29 %
Sample2	10X	5X	20X	28.57 %	14.29 %	57.14 %
Sample3	10X	5X	(Not Selected)	66.67 %	33.33 %	0 %
Sample4	10X	(Not Selected)	5X	66.67 %	0 %	33.33 %
Sample5	20X	10X	5X	57.14 %	28.57 %	14.29 %
Sample6	20X	5X	10X	57.14 %	14.29 %	28.57 %
Sample7	20X	5X	(Not Selected)	80 %	20 %	0 %
Sample8	20X	(Not Selected)	5X	80 %	0 %	20 %
Sample9	5X	10X	20X	14.29 %	28.57 %	57.14 %
Sample10	5X	10X	(Not Selected)	33.33 %	66.67 %	0.00 %
Sample11	5X	20X	10X	14.29 %	57.14 %	28.57 %
Sample12	5X	20X	(Not Selected)	20 %	80 %	0 %
Sample13	5X	5X	5X	33.33 %	33.33 %	33.33 %
Sample14	5X	5X	(Not Selected)	50.00 %	50.00 %	0 %
Sample15	5X	(Not Selected)	10X	33.33 %	0 %	66.67 %
Sample16	5X	(Not Selected)	20X	20 %	0 %	80 %
Sample17	5X	(Not Selected)	5X	50.00 %	0 %	50.00 %
Sample18	5X	(Not Selected)	(Not Selected)	100 %	0 %	0 %
Sample19	(Not Selected)	10X	5X	0 %	66.67 %	33.33 %
Sample20	(Not Selected)	20X	5X	0 %	80 %	20 %
Sample21	(Not Selected)	5X	10X	0 %	33.33 %	66.67 %
Sample22	(Not Selected)	5X	20X	0 %	20 %	80 %
Sample23	(Not Selected)	5X	5X	0 %	50.00 %	50.00 %
Sample24	(Not Selected)	5X	(Not Selected)	0 %	100 %	0 %
Sample25	(Not Selected)	(Not Selected)	5X	0 %	0 %	100 %

### 2.2 Genome Sequencing and SNP identification

The SNPs and the corresponding allele frequencies of simulated samples were identified and estimated in four steps. First, raw DNA-seq reads were trimmed using Trimmomatic (version 0.32) (Bolger et al., 2014) to remove adapter sequences for improving the sequencing depth. Second, the sequence alignment procedure aligned the trimmed DNA-seq reads to the corresponding reference genome using the alignment tool, Bwa (Li, 2013), and produced the alignment results in bam (Li et al., 2009) format. Third, SNPs and the corresponding allele frequencies were identified and estimated using the variant calling tool, gatk-haplotype (McKenna et al., 2010). Finally, we performed joint genotyping across all samples for each SNP identified in at least one sample to try to fill in any missing SNPs and allele frequency information. The bcbio-nextgen pipeline (v1.2.9) (Chapman et al., 2021) that includes the alignment and variant calling tools was used for sequence alignment, SNP identification, and joint genotyping. The analysis type parameter for specifying the task of the pipeline is set as “variants” which indicates the SNP identification task, and all other parameters in the pipeline are set as default for processing the DNA-seq data.

### 2.3 Reference SNP allele frequency profile preparation

The SNP allele frequency data of the selected three cow breeds and three cacao varieties were curated from the Bovine Genome Variation database (BGVD) (Chen et al., 2020) and the dataset published by Nieves-Orduña et al. (Nieves-Orduña et al., 2024), respectively. Because cacao data provided the genotypes rather than the allele frequencies, the conversion is applied so that the homozygous reference genotype, heterozygous genotype, and homozygous alternative genotype were converted to the allele frequency value of 0, 0.5, and 1, respectively.

### 2.4 Breed or variety composition ratio estimation using NNLS

Breed or variety composition ratios of simulated samples were estimated by solving a linear combination of reference allele frequency profiles of selected breeds or varieties to match each sample's observed SNP allele frequencies. We applied a non-negative least squares (NNLS) solver (nnls package (version 1.6) (Mullen and Stokkum, 2024) in R environment (version 4.2.0) (R Core Team, 2022)) to find the set of non-negative breed or variety composition ratios that best reconstruct each sample's allele frequency profile, with one additional condition to ensure the sum of the breed or variety composition ratio is close to 100 % (Fig. 1B). The composition ratio estimation performance was then assessed by comparing the estimated ratios to the known true ratios. We evaluated performance using several metrics, including absolute estimation error, binary classification metrics (recall, precision, F1-score, specificity) for detecting the presence of each breed or variety in a sample, and multiclass classification accuracy for identifying the majority breed or variety in each sample (Fig. 1C). To examine the robustness of the method under background noise, we introduced varying levels of noise by mixing each sample's observed SNP allele frequencies with randomly permuted versions of the same data. We tested noise ratios ranging from 0.0 to 1.0 (at intervals of 0.1; where 0 indicates no noise and 1 represents fully randomized noise input), performing 100 random permutations per noise level, and recorded the average estimation performance across replicates.

### 2.5 Comparison of estimation performance for different input SNP numbers

We also examined how the number of SNP markers affects composition ratio estimation performance by subsampling different numbers of markers (10, 50, 10^2^, 10^3^, 10^4^, 10^5^, and 10^6^). For the cow breed composition ratio estimation case, different numbers of SNP markers were randomly selected from the SNPs that were both detected in simulated samples and found in the reference allele frequency dataset. For the cow breed dataset, we randomly selected SNP markers from those presented in both the simulated samples and the reference dataset. For the cacao variety dataset, due to the limited number of reference SNPs, we instead randomly selected SNPs detected in the simulated pure-variety samples (Samples 23, 24, and 25 in Table 2) and used their estimated allele frequencies as reference profiles for composition estimation. Each random selection was repeated 100 times for each marker set size, and the average performance was recorded.

## 3 Results

### 3.1 SNP markers for breed or variety composition ratio estimation

**Fig. 2:**
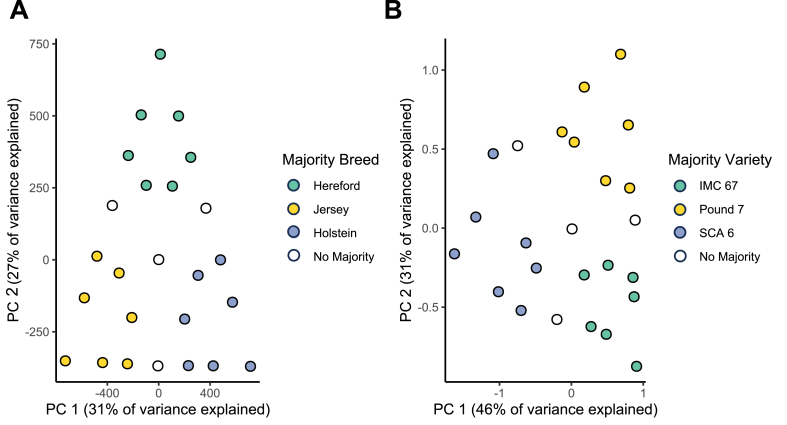
Principal Component Analysis (PCA) of SNP allele frequency profiles. **A.** PCA plot of the allele frequencies of 2.8 million SNPs from simulated cow breed mixture samples, color-coded by the majority breed. **B.** PCA plot of the allele frequencies of 11 SNPs from simulated cacao variety mixture samples, color-coded by the majority variety.

We first identified SNP markers that were both found in the reference allele frequency datasets and detected in the simulated samples (Fig. S1). These SNP markers can be used for estimating breed or variety composition ratios. For the cow breed composition ratio estimation case, the reference allele frequency dataset from the BGVD database contains approximately 37.7 million SNPs across 54 cattle breeds (including Hereford, Jersey, and Holstein). Of these, 3,005,444 of them were detected in our simulated samples. These three selected breeds have distinct SNP allele frequency patterns, which enable breed composition estimation (Fig. 2A, Fig. S2A).

For the cacao variety composition ratio estimation case, the available SNPs in the reference allele frequency data were much more limited. Only 36 SNPs across three selected cacao varieties (IMC 67, Pound 7, and SCA 6) were reported in the reference allele frequency dataset from the study by Nieves-Orduña et al. (Nieves-Orduña et al., 2024). Despite the small marker set and some discrepancies of allele frequencies between the reference genome sequence and the published genotypes, 11 SNPs exhibited variety-specific allele frequencies that make composition estimation feasible (Fig. 2B). For example, only the SCA 6 variety has high allele frequencies for two SNPs (9:8066239 (A/G) and 10:936105 (A/G)), and only the Pound 7 variety has a high allele frequency for the SNP 8:5909121 (T/C) (Fig. S2B).

### 3.2 Breed or variety composition ratio estimation in Simulated Samples

**Fig. 3:**
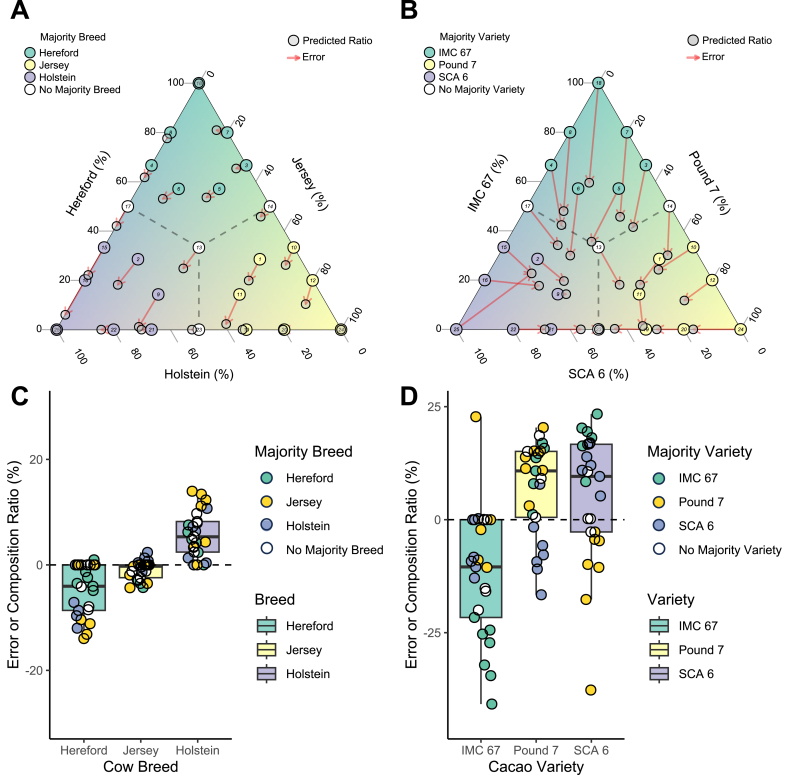
Composition ratio estimation results for simulated samples. **A.** Actual vs. predicted composition ratios for the 25 simulated cow breed mixture samples. **B.** Actual vs. predicted composition ratios for the 25 simulated cacao variety mixture samples. **C.** Estimation error (the difference between actual and predicted composition ratios) for each of the three cow breeds across the 25 samples. **D.** Estimation error for each of the three cacao varieties across the 25 samples.

**Fig. 4:**
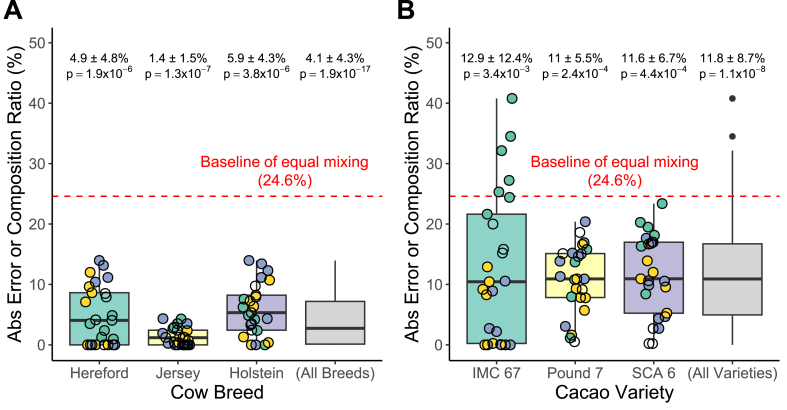
Absolute composition ratio estimation errors compared to baseline with p-values (one-tailed *t*-test). **A.** Absolute errors for each of the three cow breeds across 25 samples. **B.** Absolute errors for each of the three cacao varieties across 25 samples. The red dashed line indicates the baseline error assuming an equal one-third composition of all three breeds or varieties in every sample.

The estimated breed and variety composition ratios matched the actual values across the simulated samples (Fig. 3A and B). For the cow breed composition ratio estimation case, the tool tended to slightly underestimate the Hereford composition ratio and overestimate the Holstein composition ratio, with errors of approximately ±10 % (Fig. 3C). For the cacao variety composition ratio estimation case, errors were larger (around ±25 %) (Fig. 3D), with the tool underestimating the IMC 67 composition ratio and overestimating Pound 7 and SCA 6 composition ratios. However, the tool's estimates had significantly lower average absolute error than a naive baseline that assumes each sample is equally mixed with three different breeds or varieties (4.1 % vs 24.6 % for cows, p-value = 1.9 × 10^−17^; and 11.8 % vs 24.6 % for cacao, p-value = 1.1 × 10^−8^) (Fig. 4). Furthermore, the method maintained superior performance over the baseline even with noise ratios as high as 0.8 for cows and 0.6 for cacao (Fig. S3A and S3B). These results demonstrated that SNP allele frequency information can be used to infer breed and variety composition ratios.

### 3.3 Breed or variety presence detection and majority breed or variety identification

**Fig. 5:**
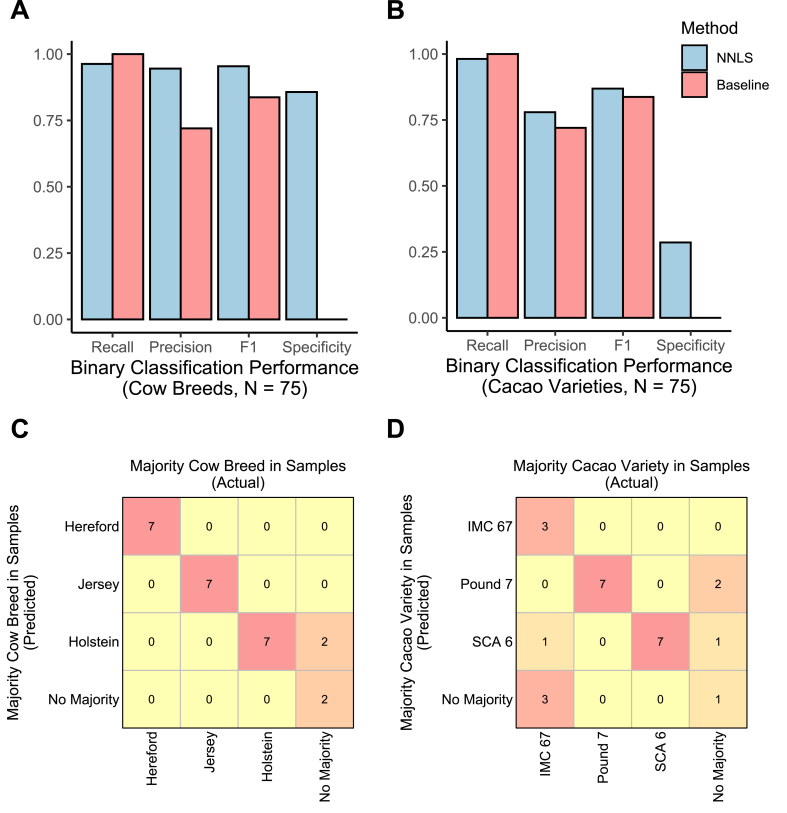
Classification performance based on estimated composition ratios. This figure summarizes binary classification (detected or not detected of each breed or variety) and multiclass classification (majority breed or variety identification) outcomes. **A.** Binary classification performance (recall, precision, F1, specificity) for detecting each breed in the cow dataset. **B.** Binary classification performance for detecting each variety in the cacao dataset. **C.** Multiclass classification result, shown as a confusion matrix for identifying the majority cow breed in samples. (the actual majority breed vs. the predicted majority breed), **D.** Multiple classification results, shown as a confusion matrix for identifying the majority cacao variety in samples.

In addition to estimating composition ratios, the tool can also detect breeds or varieties and identify the majority breed or variety in a sample. We treated the detection of three selected breeds or varieties as separate binary classification tasks (one for each breed or variety) per sample. Using a 5 % composition ratio threshold to define the existence of the breeds or varieties, we computed recall, precision, F1, and specificity by comparing the tool's estimation results to the ground truth for three breeds or varieties in all 25 samples (75 decisions in total). Fig. 5A and B show the binary classification performance for the cow and cacao datasets, respectively. Compared to a baseline that assumes every sample contains all three breeds or varieties, the tool achieved notably higher precision and F1 scores in the cow dataset, and higher specificity in both datasets. Furthermore, the tool maintained superior F1 scores under moderate noise, achieving better performance than the baseline at noise ratios of 0.5 and 0.6 for the cow and cacao datasets, respectively (Fig. S3C and S3D).

Identifying the majority breed or variety in each sample is also an important application of the tool, and it can be treated as a multiclass classification problem. The tool correctly identified the majority breed in 23 of 25 cow samples (92 %) and 18 of 25 cacao samples (72 %) (Fig. 5C and D). The performance is significantly better than the performance of the baseline that guesses one breed or variety as the majority with an accuracy of 28 % even when evaluated under high noise conditions (noise ratio = 0.8) for both cow and cacao datasets (Fig. S3E and S3F). The results show that the tool can determine the existence of specific breeds or varieties and the majority breed or variety in samples.

### 3.4 The number of SNP markers affects the composition ratio estimation performance

We found that increasing the number of SNP markers generally improved composition ratio estimation performance. Using more SNP markers led to lower absolute error in the estimated composition ratios, higher F1 scores in the binary classification for determining the existence of breeds or varieties, and higher accuracy for identifying the majority breed or variety. This trend was most significant when the SNP count was below about 1,000, as performance increased with more SNP markers (Fig. S4). The result showed that incorporating more SNP allele frequency data allows the tool to achieve better composition ratio estimation performance.

## 4 Discussion

This study demonstrated the feasibility of estimating breed or variety composition ratios in food samples containing mixtures of breeds or varieties based on the SNP allele frequencies of the reference breeds or varieties. Although prior studies have leveraged DNA sequencing to estimate species-level composition in food products (e.g., identifying proportions of meat species in sausages) (Ripp et al., 2014; Kobus et al., 2020; Hellmann et al., 2020) or traced ancestry using SNP data (Alexander and Lange, 2011; Wang et al., 2020; He et al., 2018), to our knowledge, no published work has directly estimated intra-species composition ratios—such as cow breed or cacao variety contributions—based solely on SNP allele frequencies. Our non-negative least squares (NNLS)-based approach directly computed the breed or variety composition ratio of a sample without any prior training, given reference allele frequency data for the candidate breeds or varieties. The results showed that our method outperformed the baseline with lower estimation errors, higher F1 scores in verifying whether a specific breed or variety is present, and higher accuracies in determining the dominant breed or variety in food samples.

A key challenge in this approach is the genetic variability within breeds or varieties due to crossbreeding and natural genetic diversity. For instance, the BGVD dataset showed considerable SNP variability within cow breeds, and we also observed inconsistencies between reference genome annotations and published SNP data for cacao varieties. At SNP position 5:40156015 (A/T), the genotypes in the reference genomes of IMC 67, Pound 7, and SCA 6 were A, A, and T, respectively. However, published SNP datasets reported TT, AA, and AT genotypes for IMC 67, SCA 6, and Pound 7, respectively. Such inconsistencies may lead to overestimation of minority varieties and underestimation of the dominant variety. Nevertheless, increasing the amount of reference data can mitigate these issues. As more individuals are genotyped and more breed-specific SNP markers are identified, the reference allele frequency profiles become more distinctive for each breed or variety, which can improve the composition ratio estimation performance (Fig. S4). Consistently, compared to the cacao variety composition ratio estimation case based on limited SNP markers, lower estimation errors were observed for estimating cow breed composition ratios based on a large number of SNP markers. In addition, the experiment for comparing the performance of composition ratio estimation based on varying SNP marker set sizes also showed that SNP marker set size is positively correlated with the performance.

In addition to genetic variability, real-world applications must account for noise sources such as sequencing errors, sample degradation, alignment artifacts, and potential contamination from other species in food samples. While these challenges can be partially addressed through improved experimental protocols, contamination DNA filtering tools (e.g., Kraken (Wood and Salzberg, 2014)), and multiple species screening tools for ensuring no other species in food samples (e.g., All-Food-Seq (Ripp et al., 2014)), we systematically tested robustness by introducing noise with controlled noise ratios into the allele frequency profiles. Even at a noise level of 0.5, our method maintained superior performance over a naïve baseline in terms of lower average absolute error, higher F1 scores, and improved majority classification accuracy (Fig. S3). The results indicated the robustness of our method and implied the possibility of applying this method to real food samples.

Because the NNLS method requires no model training and is computationally efficient, the tool can easily incorporate new SNP markers or be extended to additional species. In practice, this means the composition ratio estimates can be quickly updated as new reference data become available, and the method can be applied to any scenario where breed or variety allele frequency references exist. Potential applications include food authenticity testing (e.g., confirming that a dairy product contains a certain percentage of milk from the Jersey breed, which has a higher cost compared to the Holstein breed (Olthof et al., 2023)) and research on how breed composition influences product properties. For instance, the estimated breed or variety ratios could be used as features to study correlations with milk or wine chemical profiles, flavor attributes, or nutritional content. These examples highlight the practical value of our approach for the food industry and food science research.

## Author statement

The contributions of authors are listed as follows: ChengEn Tan: Data curation, Methodology, Writing, and Analysis; Ilias tagkopoulos: Conceptualization, Methodology, Funding, Administration, Writing, and Supervision. This work was supported by the United States Department of Agriculture-National Institute of Food and Agriculture AI Institute for Next Generation Food Systems (AIFS), USDA-NIFA award number 2020–67021-32855. The authors would like to thank Eve Pollet and Dairy Management Inc. for the helpful discussions on milk chemical composition and dairy product workflows.

## Declaration of generative AI and AI-assisted technologies in the writing process

Statement: During the preparation of this work the authors used ChatGPT 4o - deep research in order to improve readability and language (all contents before the refinement including manuscript, supplementary materials, and figures, are prepared by authors without using the generative AI). After using this tool/service, the authors reviewed and edited the content as needed and took full responsibility for the content of the published article.

## Declaration of competing interest

The authors declare that they have no known competing financial interests or personal relationships that could have appeared to influence the work reported in this paper.
