# Supplementary material for: Single nucleotide polymorphism information estimates breed and variety composition ratio in food
Source: Curr Res Food Sci. 2026 Jan 13;12:101312. doi: 10.1016/j.crfs.2026.101312 (PMC12856865; doi:10.1016/j.crfs.2026.101312)
Supplement: Multimedia component 1 [file mmc1.docx]

Single Nucleotide Polymorphism Information Estimates Breed and Variety Composition Ratio in Food

Cheng-En Tan^1,2,3^ and Ilias Tagkopoulos^1,2,3*^

*^1^Department of Computer Science, University of California, Davis, Davis, California, 95616 United States*

*^2^Genome Center, University of California, Davis, Davis, California, 95616 United States*

*^3^USDA/NSF AI Institute for Next Generation Food Systems (AIFS), University of California, Davis, Davis, California, 95616 United States*

^*^Email: itagkopoulos@ucdavis.edu

**SUPPLEMENTARY INFORMATION**

Supplementary Figures


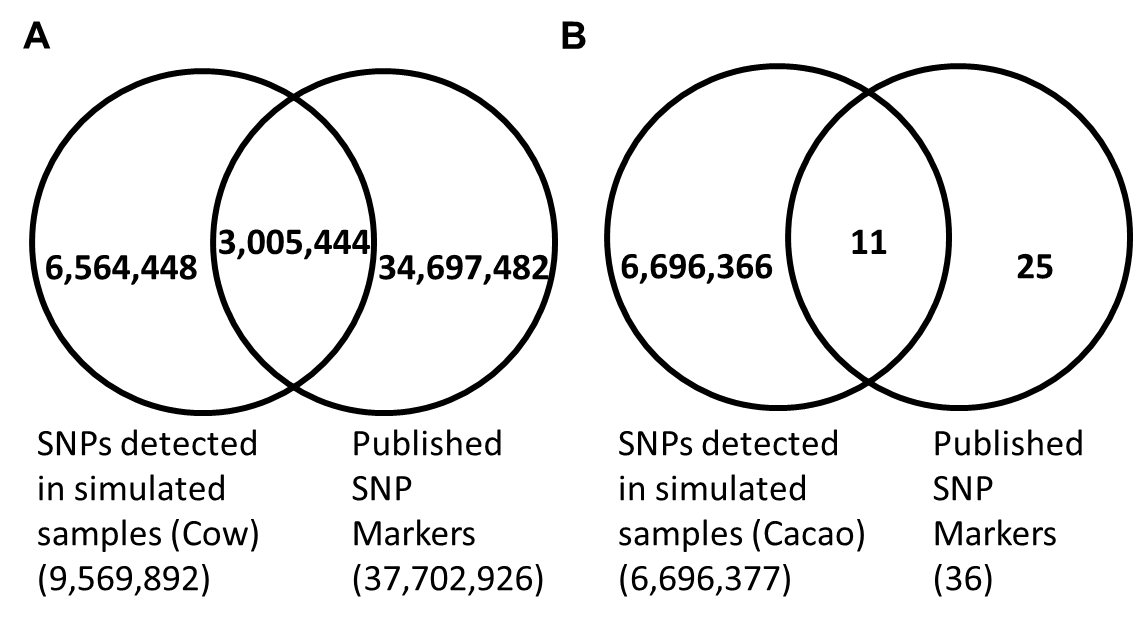


**Figure S1.** The SNPs detected in simulated samples, published SNP markers, and the intersection of them for **A.** Cow breed mixture dataset and **B.** Cacao variety mixture dataset.


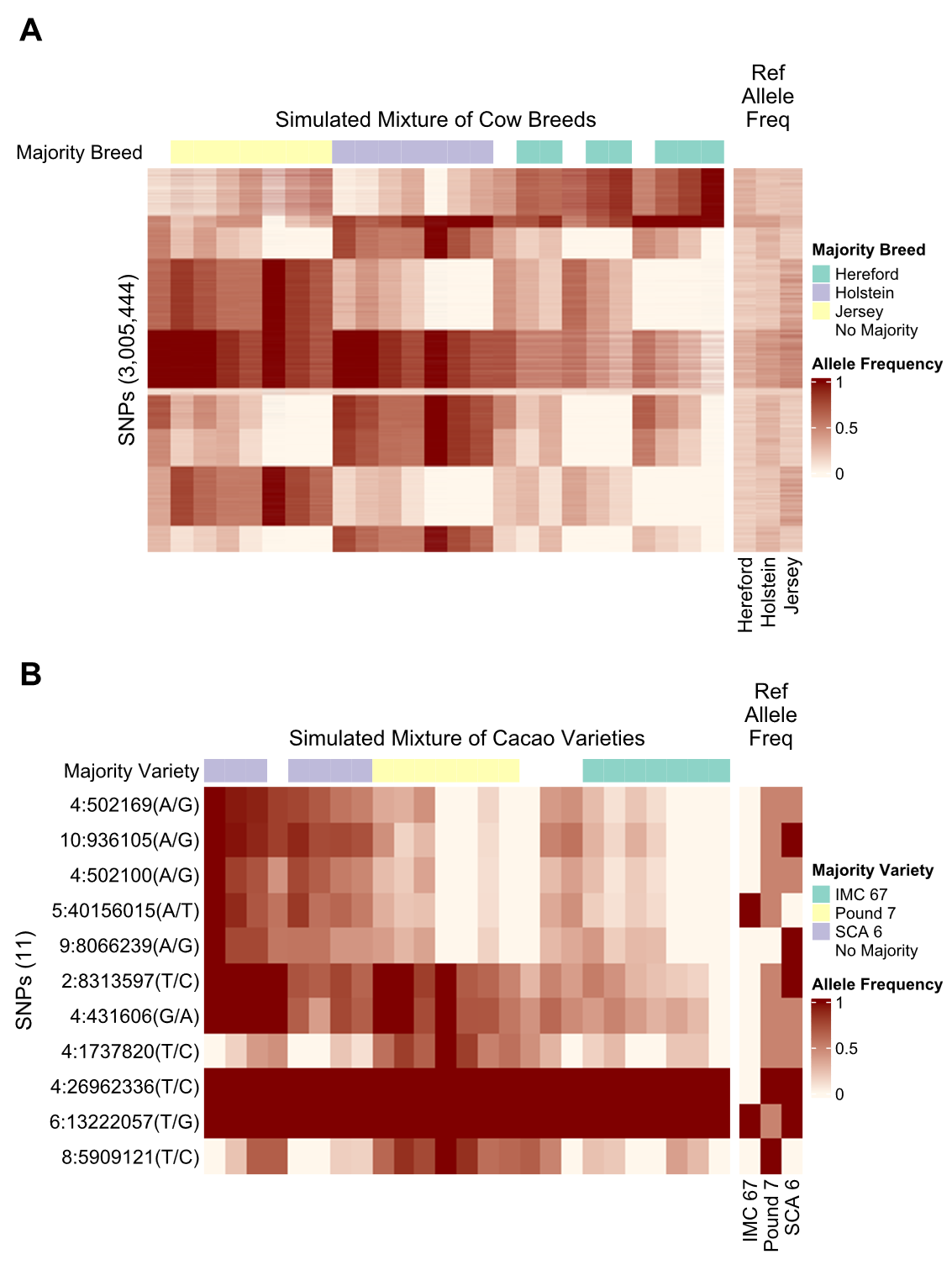


**Figure S2.** SNP allele frequency profiles of simulated samples versus references. **A.** SNP allele frequencies in simulated cow breed mixture samples compared to the allele frequencies of the three reference cow breeds (Hereford, Jersey, Holstein). **B.** SNP allele frequencies in simulated cacao variety mixture samples compared to the allele frequencies of the three reference cacao varieties (IMC 67, POUND 7, SCA 6)


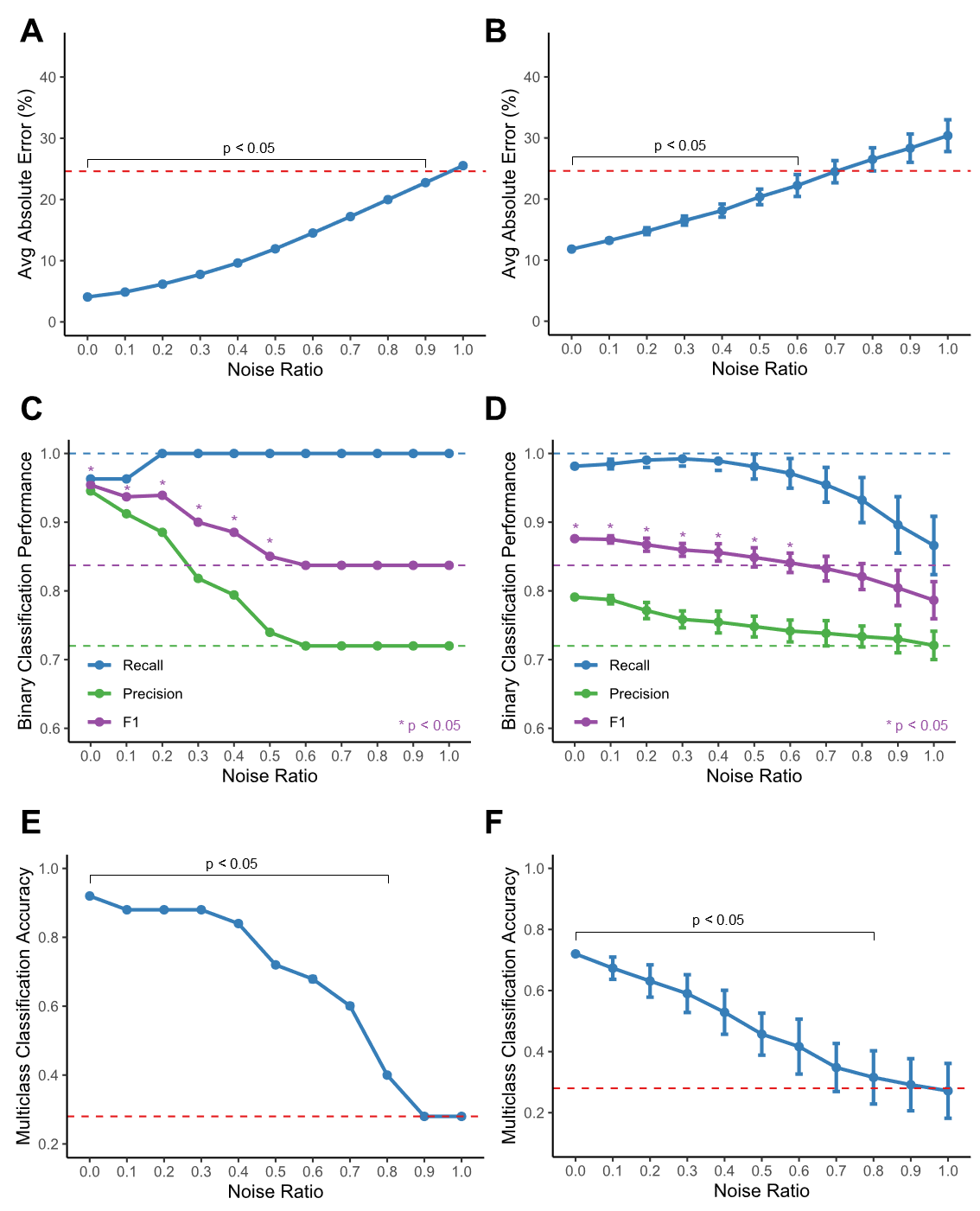


**Figure S3.** Breed and variety composition ratio estimation performance based on different noise ratios. **A.** Average absolute estimation error of the cow breed mixture dataset with the absolute estimation error of the baseline (the red dashed line) that assumed that all samples are equally mixed with three breeds, **B.** Average absolute estimation error of the cacao dataset with the baseline, **C.** Binary classification performance (recall, precision, F1-score) for detecting specific cow breeds in samples, compared to the baseline (the dashed line) that assumed that all samples contain all breeds, **D.** Binary classification performance for detecting cacao varieties with the baseline performance. **E.** Multiclass classification accuracy for identifying the majority breed in cow samples, with the accuracy of the baseline (dashed line) assuming one of the selected breeds is the majority in all samples. **F.** Multiclass classification accuracy for identifying the majority variety in cacao samples.


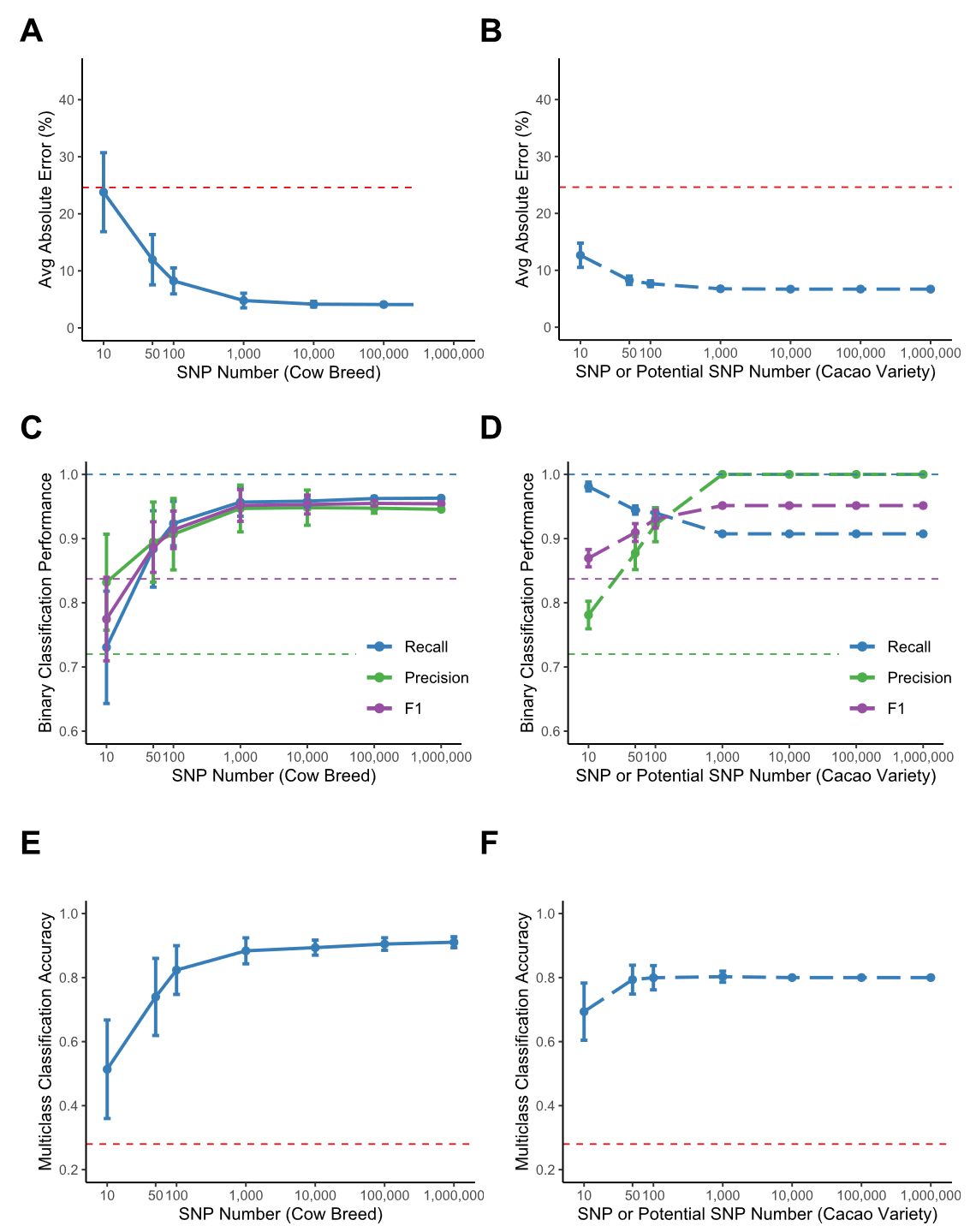


**Figure S4.** Breed and variety composition ratio estimation performance based on the different numbers of SNPs. **A.** Average absolute estimation error of the cow breed mixture dataset with the absolute estimation error of the baseline (the red dashed line) that assumed that all samples are equally mixed with three breeds, **B.** Average absolute estimation error of the cacao dataset with the baseline, **C.** Binary classification performance (recall, precision, F1-score) for detecting specific cow breeds in samples, compared to the baseline (the dashed line) that assumed that all samples contain all breeds, **D.** Binary classification performance for detecting cacao varieties with the baseline performance. **E.** Multiclass classification accuracy for identifying the majority breed in cow samples, with the accuracy of the baseline (dashed line) assuming one of the selected breeds is the majority in all samples. **F.** Multiclass classification accuracy for identifying the majority variety in cacao samples.
